# Rapid and simple isolation of precartilaginous stem cells: A novel approach without immunomagnetic bead sorting

**DOI:** 10.1016/j.heliyon.2024.e32617

**Published:** 2024-06-06

**Authors:** Song Chen, Qingshuang Zhang, Dianhua Huang, Ran Lin, Shunyou Chen

**Affiliations:** a Department of Orthopedics, Fuzhou Second General Hospital, 47th Shangteng Road of Cangshan District, Fuzhou, 350007, Fujian Province, China; b Fujian University of Traditional Chinese Medicine, No. 1, Qiuyang Road, Shangjie Town, Minhou County, Fuzhou, 350108, Fujian Province, China

**Keywords:** Precartilaginous stem cells, Cell isolation, Immunomagnetic bead sorting, FGFR-3, PCNA

## Abstract

**Objective:**

The present investigation was designed to devise a rapid and straightforward technique for the isolation of rat precartilaginous stem cells (PCSCs) that eschews the use of immunomagnetic bead sorting.

**Method:**

Rat neonates within 24 h of birth were selected for this study. Microsurgical techniques were used to harvest the femur, tibia, and the musculature of the knee joint. The ring of LaCroix between the metaphysis of the femur and the epiphysis was excised and divided into fragments of approximately 1 mm³. Tissue sections were cultured in Dulbecco's modified Eagle's medium(DMEM)/F12 medium supplemented with 20 % fetal bovine serum and 1 % penicillin-streptomycin. Cell digestion and passaging were performed using trypsin when cells reached 70%–80 % confluence. Third-generation cells underwent immunofluorescence staining and flow cytometry to evaluate fibroblast growth factor receptor-3(FGFR-3) and proliferating cell nuclear antigen(PCNA) expression, while β-galactosidase staining was used to determine cellular senescence.

**Results:**

Within two days of isolation, numerous short spindle-shaped cells exhibiting distinct refractive properties were observed around the tissue fragments. These cells began to proliferate within 2–3 days and displayed ample cytoplasm. Adherent cells adopted various morphologies, including angular, triangular, and elongated spindles. By the fifth day, more than 80 % of the culture dish surface was covered with elongated cells, with some arranged in patterns reminiscent of whirlpools. Significant FGFR-3 and PCNA expression was confirmed via immunofluorescence in the third-generation cells. Additionally, flow cytometry identified that the proportion of cells positive for FGFR-3 and PCNA exceeded 98 %. Notably, the cells preserved their proliferative capacity through nine passages in vitro, with a marginal proportion showing senescence as indicated by β-galactosidase staining alone.

**Conclusion:**

The developed tissue adherence protocol was used to successfully isolate PCSCs with positive FGFR-3 and PCNA expression, rendering the immunomagnetic bead sorting superfluous. The expression of FGFR-3 and PCNA in the isolated cells persisted through the ninth passage in vitro with minimal senescence.

## Highlights

• Unveils a novel, low-cost PCSC isolation method bypassing immunomagnetic sorting.
• Demonstrates successful isolation of FGFR-3 and PCNA positive PCSCs.
• Maintains high expression of cellular markers FGFR-3 and PCNA up to the 10th passage in vitro.
• Provides an alternative, cost-effective method for isolating PCSCs expressing FGFR-3 and PCNA.

## Abbreviations

FGFR-3: Fibroblast growth factor receptor-3
PCNA: Proliferating cell nuclear antigen
PCSCs: Precartilaginous stem cell

## 1 Introduction

Despite the increasing volume of research in recent years, the limited self-repair capability of cartilage remains a paramount issue in orthopedic medicine [1,2]. Stem cells, such as those derived from the umbilical cords [3,4], adipose tissue [5], and bone marrow [6], are extensively used in cartilage repair because of their trilineage differentiation capability. The quest for an optimal restorative solution for cartilage defects continues, as the current therapeutic strategies fall short of the ideal. A thorough understanding of the differentiation mechanisms associated with bone and cartilage is crucial within the domain of clinical tissue engineering, which aims to achieve effective cartilage repair. The milestone discovery of precartilaginous stem cells (PCSCs) by Robinson et al., in 1999 paved new pathways for bone development studies and yielded essential seed cells for tissue engineering applications [7]. Their seminal work identified fibroblast growth factor receptor-3 (FGFR-3) as a definitive marker of PCSCs and revealed that these cells to expressed high levels of proliferating cell nuclear antigen (PCNA), indicative of a substantial proliferative capacity. This is in stark contrast to mature chondrocytes and hypertrophic chondrocytes that lack FGFR-3 and PCNA expression [8]. PCSCs are deemed superior for cartilage repair because of their higher proliferative abilities, more consistent chondrogenic phenotype, reduced immunogenicity, and lower propensity for hypertrophic differentiation than stem cells from bone marrow, adipose tissue, or umbilical cords [9]. However, the procurement of a significant number of phenotypically consistent cartilage stem cells in vitro remains a daunting challenge, as differentiation is typically initiated by the fifth generation. The development of effective strategies for obtaining the requisite seed cells for tissue engineering is a challenge that remains to be unresolved, both domestically and internationally.

Therefore, PCSCs play an indispensable role in cartilage repair. These cells harbor considerable promise as seed cells for the amelioration of cartilage defects [10,11]. However, traditional PCSCs isolation methodologies, mainly enzymatic digestion followed by immunomagnetic bead sorting, present substantial challenges regarding complexity, efficiency, and the risk of contamination [12,13].

In this study, we proposed an innovative tissue adherence strategy for isolating of highly pure PCSCs, thereby circumventing the need for immunomagnetic bead sorting. This technique was used to successfully isolated PCSCs that were positive for FGFR-3 and PCNA, demonstrating their potent proliferative potential.

## 2 Materials and methods

### 2.1 Animals

Two pregnant Sprague-Dawley (SD) rats were provided by the SLAC Laboratory Animal Co.,Ltd (Shanghai, China), housed and cared for in accordance with the Federation of European Laboratory Animal Science Association guidelines, and all protocols were approved by the Animal Ethics Committee of Fujian University of Traditional Chinese Medicine(3W2023035). The pregnant rats were housed in a controlled environment with a 12-h light/dark cycle (1:1 ratio), ensuring consistent periods of activity and rest. The temperature of the housing environment was maintained at 22–24 °C, and the humidity was kept at 40–60 %, which are within the optimal range for rat health.

### 2.2 Precartilaginous stem cells isolation, culture and passage

**Fig. 1:**
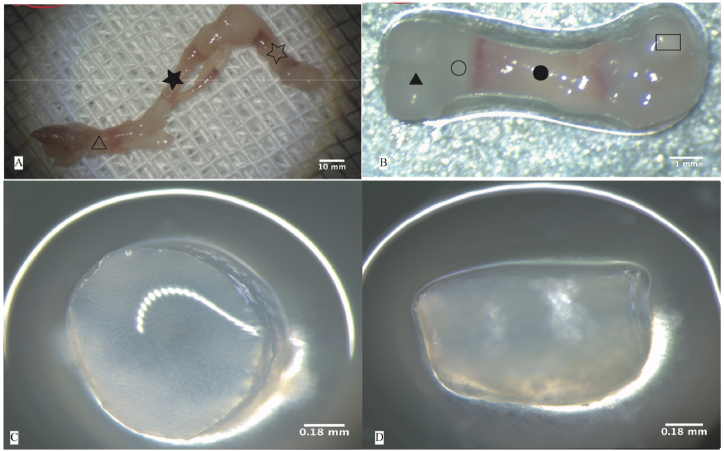
Isolation of rat epiphysis and ring of LaCroix: A) Dissection of muscles surrounding the femur and tibia (hollow star indicates femur, solid star indicates tibia, hollow triangle indicates foot). B) Intact structure of fetal rat femur (hollow rectangle indicates femoral head, solid circle indicates femoral shaft, hollow circle indicates epiphysis, solid triangle indicates distal femoral cartilage), C) Cross-sectional view of the growth plate. D) Lateral view of the growth plate.
(5) The ring of LaCroix and epiphysis were finely minced using microsurgical instruments into approximately 0.5 mm³ pieces.
(6) The finely minced tissue was evenly distributed on a 6-well plate spaced approximately 5 mm apart.
(7) The inverted 6-well plate was placed in an incubator for 1 h to promote tighter adhesion of the tissue to the culture plate.
(8) 2 mL of complete culture medium consisting of DMEM/F12 (Gibco C11330500), 20 % fetal bovine serum (Gibco 10091-148), and 1 % penicillin-streptomycin (Gibco 15140-122) was added to each well of a 6-well plate. The plate was then positioned in a humidified incubator kept at 37 °C and 5 % CO2 for 28 h, ensuring its placement in the innermost part of the incubator, minimizing disturbance or opening of the incubator whenever possible.

(1) Ten neonatal SD rats were humanely euthanized using cervical dislocation within 24h of birth.
(2) The skin was individually disinfected by immersion in 75 % alcohol.
(3) The entire hind limbs encompassing the femoral heads was excised using a surgical microscope (Leica M320 F12, Leica M125). Note: The femur is shorter than the tibia in neonatal rat pups. While dissecting the hind limbs, we ensured complete separation up to the pelvic region to fully extract both hind limbs. Inadequate extraction may compromise the femoral integrity.
(4) Delicate removal of the surrounding musculature from the femur and tibia was performed (Fig. 1-A), preserving the periosteum at the bone site. Samples were obtained from the junction of the bone shaft (red) and cartilage (colorless and transparent) (Fig. 1-B). Microscopic examination revealed the distal end epiphysis of the femur in neonatal SD rats was enveloped by a thin layer of soft tissue, identified as the ring of LaCroix (Fig. 1-C D).

### 2.3 FGFR-3, PCNA immunofluorescence and flow cytometry analysis

Third-generation of PCSCs were seeded into confocal glass-bottomed cell culture dishes (BIOFIL BDD02035）at 5 × 10^5 cells per well. After cell attachment, the PCSCs were fixed with 4 % paraformaldehyde for 15 min at room temperature. The cells were then washed three times with PBS (Gibco 2085290) for 3 min each. Subsequently, cells were blocked with 5 % donkey serum (Solarbio SL050) at room temperature for 40 min. A 0.3 % Triton X-100 solution (Solarbio T8200) was used to permeabilize the cells for 20 min. Following this, the cells were incubated overnight at 4 °C with rabbit anti-FGFR-3 antibodies (dilution 1:200, ABclonal A0404) and along with mouse anti-PCNA antibodies (Proteintech 60097-1-lg). Next, the detection of the bound primary antibodies was achieved by incubating the cells with donkey anti-rabbit IgG (H + L) Highly Cross-Adsorbed Secondary Antibody, Alexa Fluor™ 594 (Invitrogen A21207）and goat anti-mouse IgG (H + L) Secondary Antibody, Alexa Fluor® 488 conjugate Invitrogen (Invitrogen A110011）at room temperature for 1 h. The cells were then observed under a confocal microscope(Leica TCS SP8) and images were captured. PE anti-rat PCNA Antibody (Biolegend 307908), FGFR-3 antibody (Novus Biologicals NBP2-66840) were utilized for flow cytometric identification. FGFR-3 flow cytometric identification by indirect flow cytometry involves the following steps: P3 generation PCSCs were digested with trypsin (Gibico 2472358) and centrifuged at 200g for 5 min to remove the supernatant. The cells are then resuspended in PBS containing 2 % FBS, followed by another centrifugation at 200*g* for 5 min, and the supernatant is discarded. Add 50 μL of PBS containing 2 % FBS and 1.5 μL of primary antibody, then resuspend the cells. Incubate in the dark at room temperature for 30 min. Centrifuge at 200g for 5 min, discard the supernatant, and wash the cells three times with PBS containing 2 % FBS (Gibco 10091-148). Add 50 μL of PBS containing 2 % FBS and 1 μL of secondary antibody, then resuspend the cells. Incubate in the dark at room temperature for 30 min. Centrifuge at 200g for 5 min, discard the supernatant, and wash the cells three times with PBS containing 2 % FBS. Then perform flow cytometry analysis (Invitrogen Attune NxT).

### 2.4 Construction of the PCSCs growth curve chart

(1) Passage P2 PCSCs at 90 % confluence in a 12-well plate at a 1:30 ratio.
(2) Over the subsequent seven days, every 24, harvest cells from three wells of the primary culture plate using 0.25 wt% trypsin (Gibco 2472358) for complete digestion and observe and count under an inverted microscope.
(3) Calculate the average to plot a growth curve.

### 2.5 Cell senescence β‐galactosidase staining

PCSCs from passages 5 to 10 were cultivated in 6‐well plates, and cellular senescence was evaluated using β‐galactosidase staining employing the Senescence β‐Galactosidase Staining Kit (Beyotime C0602) following the manufacturer's guidelines. Briefly, cells were PBS-washed once, fixed with a fixative solution at room temperature for 15 min, PBS-washed thrice for 3 min each, and then subjected to overnight staining at 37 °C with 1000 μL of the β‐galactosidase staining solution per well. This staining solution was a composite of solutions A, B, C, and X-gal solution, as directed by the kit instructions. Subsequently, the cells were washed twice with PBS, and cell images were acquired using a phase‐contrast microscope (Novel NIB620).

## 3 Result

Observation of biological characteristics of PCSCs.

**Fig. 2:**
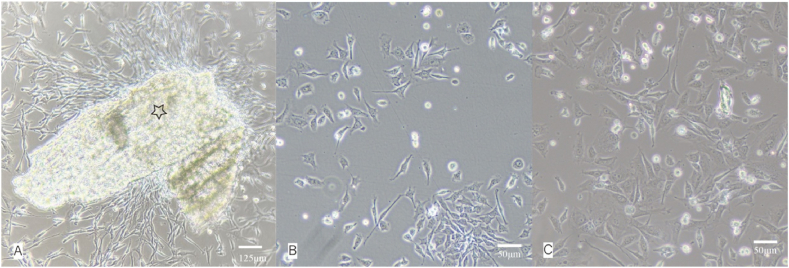
Morphology of precartilaginous stem cells (PCSCs). A）Fibrous adherent cells crawl out from tissue blocks (hollow star indicates adherent tissue block, the magnification is 10x4.). B) Morphology of P0 generation cells (the magnification is 10x10). C) Morphology of P1 generation cells (the magnification is 10x10)).

In this study, we isolated PCSCs from the rat epiphyses and the surrounding ring of LaCroix using the tissue adherence method, followed by subsequent in vitro passaging and expansion culture (Fig. 2). Morphologically, PCSCs exhibit adherent growth, notable refractivity, polygonal nuclei, limited cytoplasm, and relatively consistent cellular morphology and size. This polygonal morphology was maintained up to at least the 10th passage.

### 3.1 Immunofluorescent and flow cytometric identification of PCSCs

**Fig. 3:**
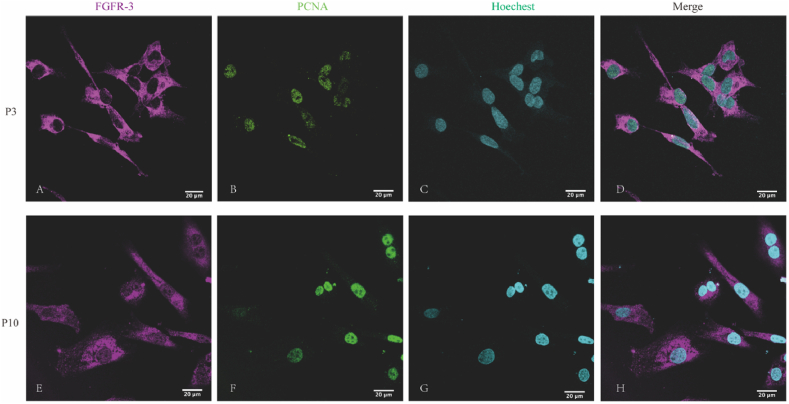
Immunofluorescence identification of P3 and P10 rat PCSCs：(A, E) immunostaining with FGFR-3, (B, F) immunostaining with PCNA, (C, G) immunostaining with hoechest, (D, G) the merged immunofluorescence image displays the co-localization of markers for FGFR-3 (magenta), PCNA (green) and hoechest(cyan) in rat PCSCs. The magnification is 10x63. (For interpretation of the references to color in this figure legend, the reader is referred to the Web version of this article.)

**Fig. 4:**
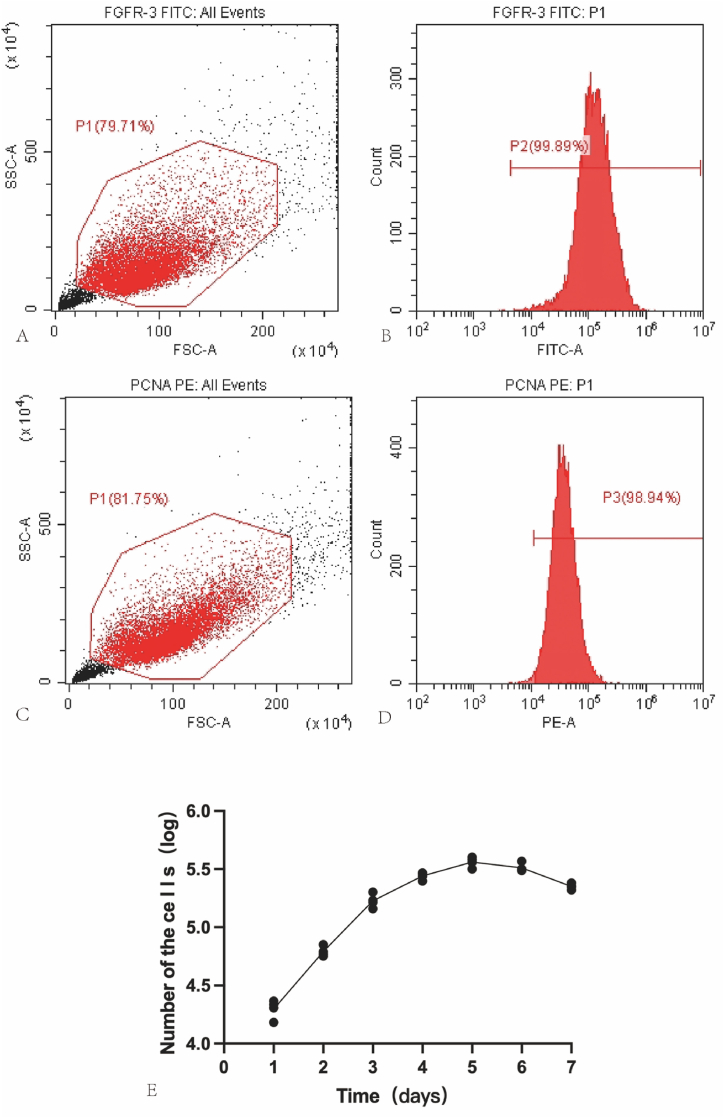
Flow cytometric identification and proliferation curve Plotting of P3 PCSCs: (A–B) Flow cytometric identification of P3 PCSCs expressing FGFR-3. (C–D) Flow cytometric identification of PCSCs expressing PCNA. (E) Proliferation curve plotting of PCSCs.

Next, immunofluoresce analysis demonstrated prominent FGFR-3 expression within the cytoplasm and cell membrane of 3rd passage PCSCs, concomitant with positive PCNA expression localized in the cell nucleus(Fig. 3A–D). To further investigate the impact of in vitro passaging on FGFR-3 and PCNA expression, we conducted additional immunofluorescence staining of cells at the tenth passage, revealing sustained high-level expression of FGFR-3 and PCNA (Fig. 3E–H). Flow cytometry identified that the proportion of cells positive for FGFR-3 and PCNA exceeded 98 % (Fig. 4A–D).

### 3.2 The growth characteristics of PCSCs

After cell inoculation, the growth rate of cells on the first day is relatively rapid. Starting on the third day, the proliferation rate of the cells gradually decreased. By the fifth day, the cells entered a plateau phase, where cell growth becomes dense and a density-dependent inhibition effect was observed. The growth curves are shown in Fig. 4 E.

### 3.3 Cell senescence β‐galactosidase staining

**Fig. 5:**
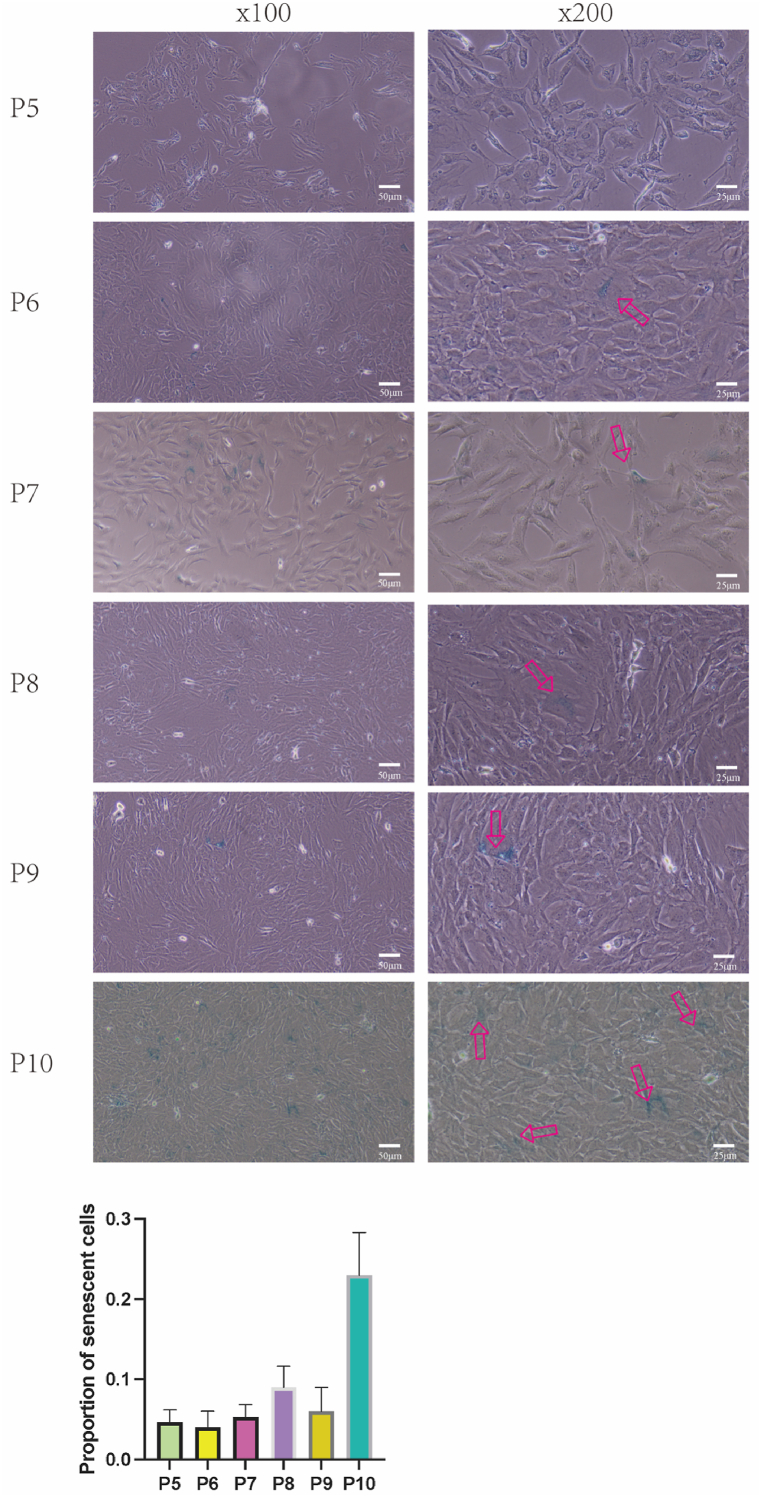
Senescence of P5 to P10 rat PCSCs assessed by β-galactosidase staining. The red arrow indicates aging cells. (For interpretation of the references to color in this figure legend, the reader is referred to the Web version of this article.)

Further assessment of cellular senescence was conducted using β-galactosidase staining. Only a minimal fraction of cells exhibited positive staining for β-galactosidase from passage 5 through 9. However, there was a noticeable increase in the number of senescent cells by the 10th passage(Fig. 5).

Indeed, in the passages 3 and 10, these cells still proliferated well, with a similar overall growth rate. We also discovered that apart from the typical blue staining observed in β-galactosidase staining, senescent cells exhibited additional morphological alterations, such as an increase in cell volume and changes in cellular morphology. These modifications typically manifest as a relatively flatter and enlarged cellular morphology compared to non-senescent cells.

## 4 Discussion

Existing methodologies for isolating PCSCs predominantly incorporate the step of immunomagnetic bead sorting [[8], [9], [10]]. Research has indicated that PCSCs procured via collagenase digestion often suffer from contamination and impurities, highlighting the need for innovative enrichment techniques for PCSCs marked by dual positivity for FGFR-3 and PCNA [11].

Immunomagnetic bead sorting and fluorescence-activated cell sorting (FACS), constitutes a conventional suite of cell sorting techniques. Immunomagnetic bead sorting is generally more cost-efficient than FACS, and the required equipment is more readily available, facilitating simpler technical operations. Nevertheless, the cost implications of immunomagnetic bead sorting are non-trivial; the majority of the studies have utilized the Mini-MACS system (Miltenyi Biotech, Bergisch Gladbach, Germany), entailing expenditures of at least 2000 US dollars. Additionally, the sorting process may lead to nonspecific binding or cell surface damage, potentially diminishing positivity rates, and consequently, the purity and precision of the isolated cells. The complexity of the immunomagnetic bead-sorting procedure and the level of technical expertise it demands mean that the success of the procedure is heavily reliant on the operator's experience and skill, which may increase the procedural complexity and difficulty. Furthermore, the mechanical or chemical manipulations to the sorting process can adversely affect cell viability and functionality, particularly in certain cell types, and may result in cellular damage or inactivation. Considering these limitations, our research aimed to develop a PCSC isolation method that circumvents the use of immunomagnetic bead sorting.

The tissue adherence method, lauded for its simplicity and cost-effectiveness, is frequently utilized for the isolation of primary cells such as mesenchymal stromal cells [12] and umbilical cord stem cells [13]. Enzymatic digestion techniques may compromise cell-surface markers or receptors, potentially leading to the loss or alteration of these markers and diminishing the accuracy and cellular purity in subsequent sorting processes [14,15]. Conversely, the tissue adherence method, which does not involve enzymatic digestion, more effectively preserves the integrity and stability of cell surface markers [16]. Moreover, enzymatic digestion may inflict mechanical or chemical damage to cells, whereas the tissue adherence method is comparatively more gentler, maintaining the integrity and viability of cells, and reducing the risk of cellular damage or inactivation, thereby facilitating the isolation of a pure population of stem cells [17].

Chondrocytes from human articular cartilage biopsy specimens did not express FGFR-3, nor do they divide or express PCNA [14]. During culture, the cells undergo dedifferentiation, leading to the restoration of FGFR-3 expression, which coincides with accelerated cell proliferation and PCNA expression [15]. Transfection with an adenoviral vector containing the gene for Escherichia coli β-galactosidase (lacZ) of PCNA, FGFR-3 double-positive cells derived from the La Croix ring demonstrated through tracing techniques that these cells migrate to osteochondral and growth plate regions on both sides of the subchondral bone plate [7]. This provides compelling evidence that PCNA and FGFR-3 double-positive cells can serve as growth plates for stem cells.

This study has the following limitations: 1. The evaluation standard for the success of PCSCs isolation was mainly based on the proportion of FGFR3 and PCNA double-positive cells, without further analysis of the chondrogenic differentiation ability of PCSCs. However, previous research by Fan et al. [9]. has already demonstrated the definitive chondrogenic potential of PCSCs. 2. In this study, β-galactosidase staining was used to assess cellular senescence, which is relatively subjective. Future research should use quantitative assessments of cellular senescence markers, such as LaminB1 [16].

In our study, we used the tissue adherence method to isolate PCSCs without resorting to immunomagnetic bead sorting. Immunofluorescence analysis of the third passage (P3) cells indicated that the vast majority of cells exhibited robust expression levels of FGFR-3 and PCNA. β-galactosidase staining revealed that PCSCs isolated through the tissue adherence method showed a small proportion of cells with signs of senescence up to the ninth in vitro passage. By passage ten, a marked increase in the number of senescent cells was observed.

## 5 Conclusion

Tissue adherence effectively isolates precartilaginous stem cells expressing FGFR-3 and PCNA, eliminating the need for immunomagnetic bead sorting during enzymatic digestion. In vitro, the expression of FGFR-3 and PCNA remained sustainable throughout the 9th passage, with a negligible proportion of cells demonstrating signs of senescence.

## Funding

This project was supported by the 10.13039/501100003392Natural Science Foundation of Fujian Province, China (Grant No. 2022J01311) Key Clinical Specialty Discipline Construction Program of Fuzhou, Fujian, P.R.C (Grant No. 20220104) Fujian Provincial Clinical Medical Research Center for First Aid and Rehabilitation in Orthopaedic Trauma (Grant No. 2020Y2014).

## Data availability statement

Researchers interested in accessing the data should submit their requests to the corresponding author, who will respond accordingly.

## CRediT authorship contribution statement

**Song Chen:** Writing – original draft, Methodology, Data curation. **Qingshuang Zhang:** Writing – original draft, Methodology, Data curation. **Dianhua Huang:** Writing – original draft, Visualization. **Ran Lin:** Project administration, Formal analysis. **Shunyou Chen:** Writing – review & editing.

## Declaration of competing interest

The authors declare that they have no known competing financial interests or personal relationships that could have appeared to influence the work reported in this paper.
